# Co-learning synaptic delays, weights and adaptation in spiking neural networks

**DOI:** 10.3389/fnins.2024.1360300

**Published:** 2024-04-12

**Authors:** Lucas Deckers, Laurens Van Damme, Werner Van Leekwijck, Ing Jyh Tsang, Steven Latré

**Affiliations:** IDLab, imec, University of Antwerp, Antwerp, Belgium

**Keywords:** spiking neural networks, synaptic delays, neuronal adaptation, co-learning, speech recognition, surrogate gradients

## Abstract

Spiking neural network (SNN) distinguish themselves from artificial neural network (ANN) because of their inherent temporal processing and spike-based computations, enabling a power-efficient implementation in neuromorphic hardware. In this study, we demonstrate that data processing with spiking neurons can be enhanced by co-learning the synaptic weights with two other biologically inspired neuronal features: (1) a set of parameters describing neuronal adaptation processes and (2) synaptic propagation delays. The former allows a spiking neuron to learn how to specifically react to incoming spikes based on its past. The trained adaptation parameters result in neuronal heterogeneity, which leads to a greater variety in available spike patterns and is also found in the brain. The latter enables to learn to explicitly correlate spike trains that are temporally distanced. Synaptic delays reflect the time an action potential requires to travel from one neuron to another. We show that each of the co-learned features separately leads to an improvement over the baseline SNN and that the combination of both leads to state-of-the-art SNN results on all speech recognition datasets investigated with a simple 2-hidden layer feed-forward network. Our SNN outperforms the benchmark ANN on the neuromorphic datasets (Spiking Heidelberg Digits and Spiking Speech Commands), even with fewer trainable parameters. On the 35-class Google Speech Commands dataset, our SNN also outperforms a GRU of similar size. Our study presents brain-inspired improvements in SNN that enable them to excel over an equivalent ANN of similar size on tasks with rich temporal dynamics.

## 1 Introduction

Spiking neural networks (SNN), seen as the third generation neural network models (Maass, 1997), have recently attracted growing attention as a low-power alternative for artificial neural networks (ANN). Unlike ANN implementations (Garćıa-Mart́ın et al., 2019), SNN can enable power-efficient processing on neuromorphic hardware such as SENeCa (Yousefzadeh et al., 2022), the Intel Loihi2 (Orchard et al., 2021), or IBM TrueNorth (DeBole et al., 2019). The main power gains can be attributed to SNN inherent event-based computations (by means of spikes) and sparsity, reducing the number of multiplications that are required. A recent study (Stöckl and Maass, 2021) illustrated these potential SNN energy savings.

Recent years have shown great progress in learning algorithms for deep SNN. Especially training SNN, based on backpropagation-through-time with surrogate gradients (Neftci et al., 2019), helped overcome the problem of the non-differentiability, which was introduced by the thresholding mechanism in a spiking neuron. These advances enabled SNN to move to deeper and more complicated model architectures using attention mechanisms (Yao et al., 2023) or transformers (Zhou et al., 2023; Zhu et al., 2023). The main issue related to SNN however persists: frequently the SNN model does not perform as well as the equivalent ANN.

In biology, researchers have found that the brain is equipped with a plethora of powers to process spike trains adequately. One of those is the axonal delay. The transmission speed of an action potential is known to depend on the myelination of the axon and thus determine the extent to which a spike is delayed (Purves et al., 2001). Furthermore, these delays are crucial in sensory processing (Orchard and Etienne-Cummings, 2014) and known to adapt during the learning process (Lin and Faber, 2002). A delay-enabled spiking network was also found to be able to compute a richer class of functions than a threshold circuit with adjustable weights (Maass and Schmitt, 1999). Another differentiator between classical ANN and the brain is the widespread heterogeneity and neuronal adaptation processes taking place. Neurons of all forms and shapes are found, enabling a wide array spike pattern processing functions (Gerstner and Kistler, 2002). Moreover, biological neurons exhibit slow dynamical processes that act at longer time scales, enabling processing of events that are temporally distanced in an implicit manner. These adaptive processes often limit the number of spikes produced.

**Figure 1:**
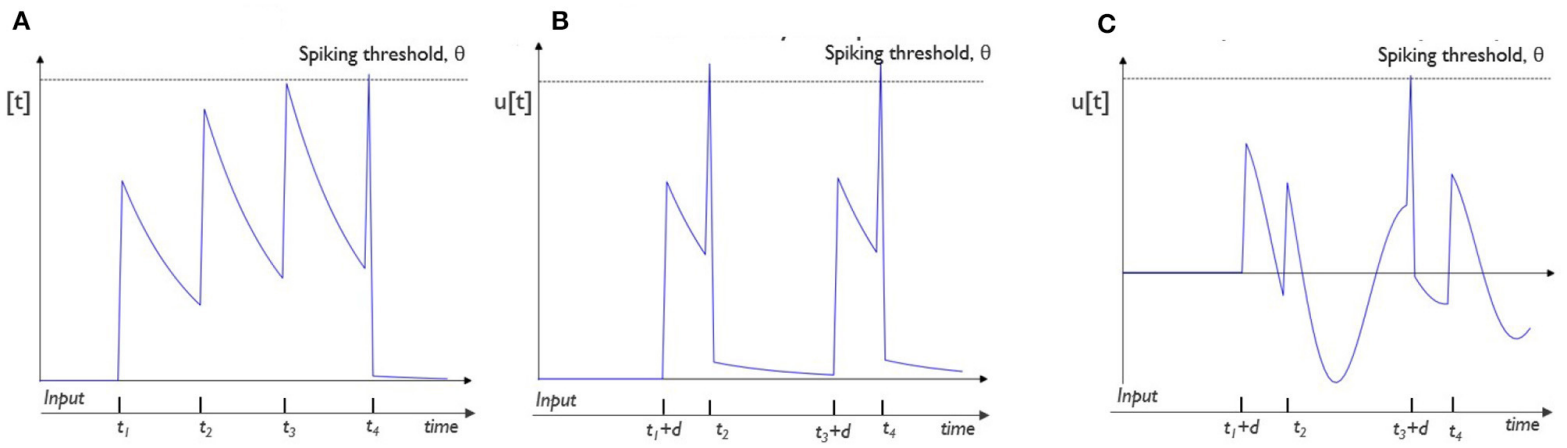
Illustration of the variety in responses for different neuron models: two input spike trains are processed by three different neurons with equal weights. Input neuron 1 spikes at (*t*_2_, *t*_4_) and input neuron 2 spikes at (*t*_1_, *t*_3_). For all neurons, we show the evolution of the membrane potential, u[t] over time in response to these input spikes: **(A)** A typical leaky-integrate-and-fire (LIF) neuron, spikes at the timestep of the last incoming spike, *t*_4_. **(B)** A LIF neuron with delayed (by *d*) input spikes from input neuron 2 produces spikes at timesteps *t*_2_ and *t*_4_. **(C)** An adaptive neuron processes delayed input spikes from input neuron and only produces a spike at timestep *t*_3_ + *d*, the latency of the spikes coming from neuron 2.

Combining the ability to optimize the weights, delays and training neuronal parameters can lead to a more diverse and possibly improved internal representation. Figure 1 shows the responses of (A) a typical leaky integrate-and-fire (LIF) neuron, (B) a neuron with delayed input spike trains, and (C) a neuron with delayed input spike trains and neuronal adaptation for a spike train of four equidistant input spikes and all equal connection weights. It can clearly be observed that the output spike patterns are vastly different. The LIF neuron (A) will always respond the same while the others provide a wider range of possible results because of the trainable delay (B) and non-linear, trainable adaptation processes (C). Both extensions show to be complementary in providing additional memory. The synaptic delay allows the neuron to explicitly correlate incoming spikes at longer timescales, whereas the adaptation implicitly alters the behavior of a neuron based on its past regime.

In this study, we present an SNN with adaptive neurons and synaptic delays that are co-optimized. Whereas, normally, as in ANN, just the synaptic weights are trained, we show that co-learning the delays and adaptation parameters individually enhance the performance of the SNN model, and that combining them even leads to state-of-the-art SNN results. The contribution of this study is summarized as follows:

1. We analyze the biologically plausible neuronal adaptation parameters and which effects parameter boundaries have on the neurons working regime as well as on the SNN performance on three speech recognition datasets.
2. We introduce a novel learning rule for synaptic delays, which accounts for temporal context.
3. We present a novel SNN model in which both synaptic weights and delays are co-optimized in collaboration with the neuronal adaptation parameters.
4. We show that the inclusion of these more complex neurons through adaptation and the addition of trainable synaptic delays for every synapse specifically leads to state-of-the-art results for spiking neural networks. The proposed SNN even outperforms its non-spiking counterpart with equivalent model size on the speech recognition problems.

## 2 Related work

### 2.1 Learning algorithms for spiking neural networks

Recently, there has been a rapid evolution in the development and progress of SNN learning paradigms. In general, either a trained ANN is converted into a rate-based SNN, aiming at minimal performance losses due to the ANN-SNN conversion (Deng and Gu, 2021; Bu et al., 2022) or the SNN is trained directly as a spiking neural network. A directly trained SNN is typically trained with backpropagation-through-time with surrogate gradients (Neftci et al., 2019; Zenke and Vogels, 2021). These surrogates are used to approach the derivative of non-differential Heaviside function, which is introduced by the spiking mechanism. A recent study went one step further in using differentiable spikes (Li et al., 2021) for temporal credit assignment. Moreover, the spike-element-wise (SEW) ResNet (Fang et al., 2021a) was proposed for training SNN without the vanishing/exploding problem introduced by surrogate gradients, paving the way for deeper SNN models.

### 2.2 Learning of neuronal parameters and neuronal heterogeneity

Another trend in SNN research is to learn the optimal distribution of the neuronal (leakage) parameters (Fang et al., 2021b; Yin et al., 2021; Rathi and Roy, 2023). Moreover, the Gated LIF neuron (Yao et al., 2022) was proposed to control the fusion of learnable membrane-related parameters. Additionally, in many studies, the heterogeneity of neurons in SNN proved to be beneficial for improved recognition performance (Perez-Nieves et al., 2021; Deckers et al., 2022; Chakraborty and Mukhopadhyay, 2023).

Different methods for including neuronal adaptation processes have been proposed. The first class contains neurons with an adaptive threshold, which is increased after every spike and exponentially decays over time (Salaj et al., 2021; Yin et al., 2021). Others (Falez et al., 2019) proposed a method for tuning the thresholds with specific target spike timestamps as an objective. In other studies (Gast et al., 2020), the adaptive thresholds were combined with a synaptic depression model, which showed to replicate both bursting and steady-state behavior. Another adaptation method is based on adaptation currents, coupling a secondary variable to the sub-threshold membrane potential and its spike activity (Brunel et al., 2003). This method was successfully formalized into the AdLIF spiking neuron model (Bittar and Garner, 2022) for SNN and was shown to outperform adaptive threshold-based models.

### 2.3 Delays in SNN

Many methods have been proposed for adapting propagation delays, inspired by spike timing dependent plasticity (Wang et al., 2013) or based on the ReSuMe learning rule (Zhang et al., 2020). A method for training per neuron axonal delays based on the SLAYER learning paradigm was proposed (Shrestha and Orchard, 2018) and extended (Sun et al., 2023b) with trainable delay caps. Recently, the effects of axonal synaptic delay learning were studied by pruning multiple delay synapses (Patiño-Saucedo et al., 2023), modeling a one-layer multinomial logistic regression with synaptic delays (Grimaldi and Perrinet, 2023) and learning delays represented trough 1D convolutions with learnable spacings (Hammouamri et al., 2023). Similarly, in order to train synaptic delays, spike trains were transformed into continuous analog, differentiable signals (Wang et al., 2019). Surprisingly, only learning the delays (Grappolini and Subramoney, 2023) showed to achieve comparable performance, only learning the weights.

## 3 Materials and methods

A spiking neural network (SNN) is a biologically inspired type of neural network, in which spikes, i.e., binary events are used to communicate between layers of spiking neurons. In this section, we elaborate on the fundamental properties of spiking neurons and the methods used in this study to train the synaptic weights, synaptic delays, and neuronal parameters in multi-layer networks of spiking neurons.

### 3.1 Spiking neurons

Spiking neurons differ from classical neurons in ANN because of their inherent time-dependent processing of data streams. Incoming spikes are multiplied by the synaptic weights and accumulated over time for every neuron. When this neuronal state, i.e., the membrane potential crosses the spiking threshold, a neuron emits a spike to a subsequent layer. The membrane potential is maintained over time and thus creates an internal memory for every individual neuron.

In this study, we use the adaptive leaky integrate-and-fire neuron (AdLIF) model (Bittar and Garner, 2022) with updated parameter boundaries. To highlight this modification, our neuron model is called the constrained AdLIF, (cAdLIF). In this model, two internal states are kept: the membrane potential and the adaptation current, which provides the neuronal adaptation. Formally, *u*[*t*], *w*[*t*], and *s*[*t*], respectively, represent the membrane potential, the adaptation current, and the presence of a spike, at time step t. In the AdLIF neuron model, there are four trainable neuronal parameters: α and β denote the leak of u[t] and w[t], respectively, while *a* and *b* describe the characteristics of the adaptation current. The adaptation current is coupled with the sub-threshold membrane potential by *a*, while *b* represents the spike-triggered adaptation. In the cAdLIF model, these *a* and *b* are constrained to be positive only. This change results in major differences when processing spikes, as discussed in Section 4.3. A neuron generates a spike at timestep t when the membrane potential crosses the firing threshold, θ, at t. Mathematically, the threshold θ is represented as a Heaviside function. The full discrete-time model, with time step size equal to 1 ms, is described in Equation (1). The trainable neuronal parameters are highlighted in bold.


(1) $$\begin{array}{l} \begin{array}{l} u\left[t\right]=\alpha u\left[t-1\right]+\left(1-\alpha \right)\left(I\left[t\right]-w\left[t-1\right]\right)-\theta s\left[t-1\right]\\ w\left[t\right]=\beta w\left[t-1\right]+\left(1-\beta \right)\text{a}u\left[t-1\right]+\text{b}s\left[t-1\right]\\ s\left[t\right]=u\left[t\right]\geq \theta \end{array} \end{array}$$

**Figure 2:**
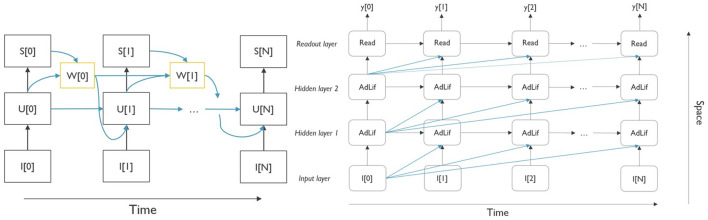
**(Left)** Computational graph of the AdLIF neuron model, unrolled over time. The yellow blocks denote the additional adaptation current parameter, which depends on the membrane potential u[t] and the spike activity, s[t] at the previous timestep. **(Right)** Two-layer fully connected architecture with readout of the output neuron membrane potential, unrolled over the time. The blue connections denote the potential delays in the network. For clarity, the delays starting from time step 1 onward were omitted.

The adaptation, implemented by means of this adaptation current *w*[*t*] is affected by both the neurons' instantaneous membrane potential and a spike-triggered fraction. This contrasts with the adaptive neuronal threshold adaptation, in which only the spiking activity is taken into account (Yin et al., 2021). This model was chosen because of its proven superior performance in comparison with a leaky integrate-and-fire (LIF) model with an adaptive neuronal threshold (Bittar and Garner, 2022). The computational graph of the neuron model, rolled out over time, is shown in Figure 2. The yellow box, *w*[*t*], represents the addition of the adaptation variable to the classic LIF neuron model, and the blue arrows, connecting the internal variables, represent the corresponding trainable neuron parameters.

### 3.2 Training multi-layer SNN

#### 3.2.1 Model architecture

In this study, the SNN consists of a simple feed-forward network with two hidden layers. Figure 2 shows the network architecture, which is rolled out over time. In general, neuron *i* in hidden layer *l* receives $I_{i}^{l}$, the pre-synaptic current, which consists of two elements, as shown in Equation (2). The feed-forward synapses from the previous layer *l*−1 have associated weights $F_{ij}^{l-1}$ and carry spikes from the same time step *t* to neuron j and the neuronal bias, $b_{i}^{l}$. These input spike trains are summed over all pre-synaptic neurons *j* = 1, ..., *N*_*l*−1_. Spike trains are represented by *s*[*t*] ∈ [0, 1]. Typically, in this type of architecture, all feed-forward are connected in an all-to-all fashion.


(2) $$\begin{array}{l} I_{i}^{l}=\overset{N_{l-1}}{\underset{j=1}{\sum }}F_{ij}^{l-1}s_{j}^{l-1}\left[t\right]+b_{i}^{l} \end{array}$$

The readout mechanism, which is used to derive the outputs of the SNN, consists of a single layer. This layer consists of neurons with infinite threshold. These neurons have no memory, and hence, the membrane potential is equal to the the weighted inputs. The output of the SNN model is the sum of the membrane potential of the output neurons over time, which is passed though a softmax layer for every timestep. The outputs are shown in Equation (3).


(3) $$\begin{array}{l} u_{out}=\underset{t}{\sum }\frac{e^{u_{out}\left[t\right]}}{\sum_{j}e^{u_{out,j}\left[t\right]}} \end{array}$$

#### 3.2.2 Training procedure

Typically, spiking neural networks (SNN) are trained via backpropagation-through-time (BPTT) with surrogate gradients (Neftci et al., 2019). In these methods, the summed membrane potential of the output neurons, see Equation (3), constitute the cross-entropy loss of the network, which is unrolled over time. The loss with respect to class c for a batch size *N* is represented as follows:


(4) $$\begin{array}{l} L_{c}=\frac{1}{N}\overset{N}{\underset{n=1}{\sum }}-log\left(\frac{e^{u_{out,c}}}{\sum_{j}e^{u_{out,j}}}\right) \end{array}$$

Based on the chain rule in error-backpropagation, the weight update for neuron *i* in the penultimate layer *l* for a sequence of T timesteps is shown in Equation (5).


(5) $$\begin{array}{l} \frac{\delta L_{c}}{\delta w^{l}}=\frac{1}{T}\overset{T}{\underset{t=1}{\sum }}\overset{t}{\underset{m=0}{\sum }}\frac{\delta L_{c}\left[t\right]}{\delta u_{out}\left[m\right]}\frac{\delta u_{out}\left[m\right]}{\delta s_{l}\left[m\right]}\frac{\delta s_{l}\left[m\right]}{\delta u_{l}\left[m\right]}\frac{\delta u_{l}\left[m\right]}{\delta w_{l}} \end{array}$$

In these methods, surrogate gradients are used to approximate the non-differentiability, which was introduced by the thresholding mechanism (Heaviside function), $\frac{\delta s\left[t\right]}{\delta u\left[t\right]}$, with a differentiable function in the backward pass of error-backpropagation. For simplicity and comparability with the previous study (Bittar and Garner, 2022), the boxcar surrogate gradient function, shown in Equation (6), was used in this study.


(6) $$\frac{\delta s[t]}{\delta u[t]}=\{\begin{array}{ll} 0.5 & \text{if }|u[t]-\theta \text{|}\leq \text{0}.\text{5}\\ 0 & \text{otherwise} \end{array}$$

### 3.3 Introducing synaptic delays

In contrast to ANNs and most other SNNs in which connections are defined by a weight parameter, in this study, connections between neurons are characterized by two synaptic parameters: a weight and a delay. The addition of synaptic delays leads to an adjusted neuronal processing model. Now, the spike train for neuron *i* from layer *l* is characterized by $I_{l}^{i}$, as shown in Equation (7). The synaptic delay can be found in $s_{j}^{l}\left[t-d_{ij}\right]$, where *d*_*ij*_ denotes the delayed spikes between neuron j and i.


(7) $$\begin{array}{l} I_{i}^{l}=\overset{N_{l-1}}{\underset{j=1}{\sum }}F_{ij}^{l-1}s_{j}^{l-1}\left[t-d_{ij}\right]+b_{i}^{l} \end{array}$$

Training of the synaptic delays is an adaptation of the SLAYER learning method (Shrestha and Orchard, 2018) for training axonal delays, which is applied to individual synapses based on the local temporal context. The delay kernel, ϵ_*d*_, is convolved with spike train s[t], to get the delayed spike kernel, $p^{l}\left[t\right]=\left(\epsilon_{d}*s^{l}\right)\left[t\right]$. Similarly to the case in which delays were equal to 0 (i.e., without delays), the derivative of the loss with respect to the synaptic weight and delays is now computed, as shown in Equations (8, 9).


(8) $$\begin{array}{l} \frac{\delta L_{c}}{\delta w^{l}}=\frac{1}{T}\overset{T}{\underset{t=1}{\sum }}\overset{t}{\underset{m=0}{\sum }}\frac{\delta L_{c}\left[t\right]}{\delta u_{out}\left[m\right]}\frac{\delta u_{out}\left[m\right]}{\delta a_{l}\left[m\right]}\frac{\delta a_{l}\left[m\right]}{\delta u_{l}\left[m\right]}\frac{\delta u_{l}\left[m\right]}{\delta w_{l}} \end{array}$$

(9) $$\begin{array}{l} \frac{\delta L_{c}}{\delta d^{l}}=\frac{1}{T}\overset{T}{\underset{t=1}{\sum }}\overset{t}{\underset{m=1}{\sum }}\frac{\delta L_{c}\left[t\right]}{\delta u_{out}\left[m\right]}\frac{\delta u_{out}\left[m\right]}{\delta p_{l}\left[m\right]}\frac{\delta p_{l}\left[m\right]}{\delta d_{l}} \end{array}$$

**Figure 3:**
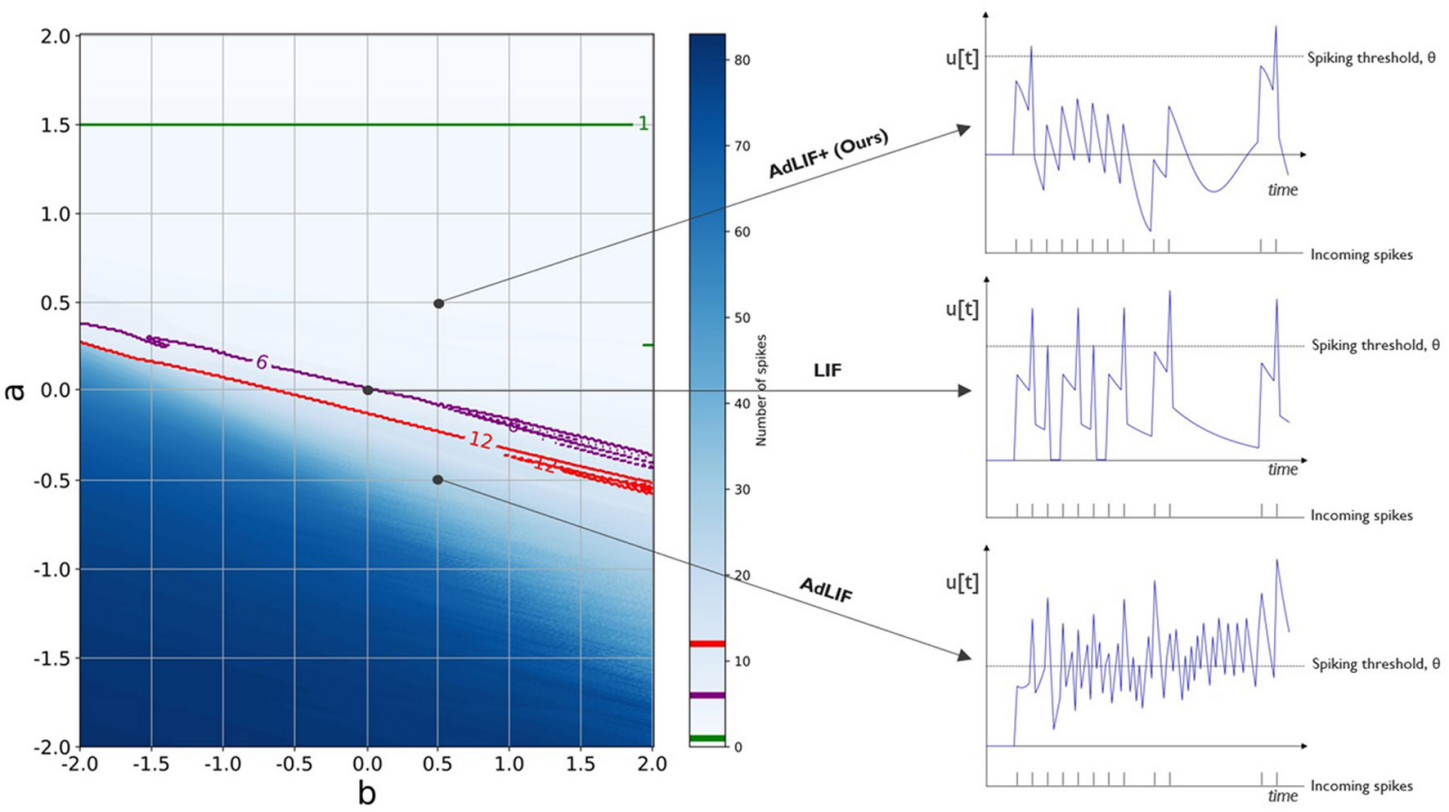
Illustration of different neuron model adaptation parametrizations and their responses to a fixed train of 12 incoming spikes. We analyze the number of output spikes and the corresponding evolution of the membrane potential u[t] over time for a range of *a* and *b* neuronal adaptation parameters. A model without adaptation, the LIF model, is found where both *a* and *b* are equal to zero. The AdLIF model, which allows both positive and negative *a*, can possibly result in an unstable spiking regime. In this case, the neuron generates more spikes than the number of incoming spikes, 12 in this example. We therefore constrained the updated neuron model, cAdLIF to remain within positive *a* and *b* boundaries. For reference, we also show the parametrizations for 6 and 1 output spikes in purple and green, respectively.

In Equation (9), $\frac{\delta p_{l}}{\delta d_{l}}$ is equal to $\frac{dp\left[t\right]}{dt}$ for every unique synapse. Contrary to the SLAYER delay learning algorithm as presented in the study by Shrestha and Orchard (2018), where the finite difference instantaneous derivative with respect to time is taken or Sun et al. (2023a) where the axonal, i.e., identical for every postsynaptic neuron, delay is implemented as a variable axonal delay module, we take the temporal context into account for every individual synapse. More precisely, $\frac{dp\left[t\right]}{dt}$ is replaced with *p*[*t* − *c*:*t* − 1] − *p*[*t* + 1:*t* + *c*] for all t timesteps per synapse. In this way, the direction of the delay updates is determined by the corresponding temporal context *c* both in the past and future of a specific timestep t and not just the direct derivative at *t*. We experimented with different ranges of temporal contexts in the learning rule for synaptic delays. To this end, we compared different values of *c*. We empirically found a width of 3 to be optimal. A detailed analysis is shown in Figure 3.

## 4 Experiments

In this section, we will elaborate on the experiments conducted for this study and the corresponding results. First, we describe the datasets and the precise setup for training the SNNs. Following this, we present an analysis of the AdLIF neuron model and, finally, show the full results.

### 4.1 Datasets

We used three common SNN benchmark speech recognition datasets: the Spiking Heidelberg Digits (SHD), the Spiking Speech Commands (SSC) (Cramer et al., 2020), and the Google Speech Commands v0.02 (GSC) dataset (Warden, 2018). The first two datasets are neuromorphic datasets, in which the original sounds were converted to spikes, spread out over 700 input channels. The GSC dataset consists of speech samples. The SHD dataset consists of German and English spoken digits (0 through 9). The SSC dataset is based on the sounds from the GSC dataset. Both the SSC and GSC datasets have 35 classes from a large group of speakers in a non-controlled environment. These larger datasets provide a more challenging speech recognition task.

Similarly to other studies, for the spiking datasets, which were zero-padded and aligned to 1 s, we binned the input data from 700 input channels to 140 channels and 100 timesteps with 10 ms bins to ensure uniformity across all samples. Regarding the GSC dataset, speech data were aligned to 1 s by padding with zeros and thereafter binned in 10 millisecond bins to generate samples of 100 timesteps. Further processing was performed with a Mel filterbank with 40 Mel filters. Since there is no predefined test set for the SHD dataset, we decided to take the average accuracy on the validation set across 10 experiments with different random seeds. Similarly, three random trials were conducted for the SSC and GSC datasets.

### 4.2 Training setup

All neuronal trainable parameters were uniformly initialized between specific boundaries and subsequently co-learned with the other model trainable parameters to reflect the neuronal heterogeneity (Perez-Nieves et al., 2021). These parameters were initialized following a uniform distribution: α ∈ [0.36, 0.96], β ∈ [0.96, 0.99], *a* ∈ [0, 1], and *b* ∈ [0, 2]. Different from the AdLIF model (Bittar and Garner, 2022), we extended the available range of the membrane potential decay parameter α and limited the dependency of the adaptation current with respect to the membrane potential, *a*, to remain positive, which showed to stabilize the neuron model for sparse input data. During the training process, all neuron parameters are clipped to remain within these boundaries. The spike threshold was fixed at 1 (dimensionless). The weights of all connections were initialized, following the default Xavier uniform distribution, and the neuronal biases were set to zero. For all hidden neurons, the initial membrane potential and adaptation current were zero-initialized.

We used the Adam optimizer (Kingma and Ba, 2014) in all experiments with an initial learning rate of 0.01 for the SHD dataset and 0.001 for the others, which is similar to the previous study (Bittar and Garner, 2022). The learning rate for the delays was always equal to 10*lr_*weigths*_. We used a simple scheduler for both the weights and delays, which decreased the learning rate by a factor of 0.7 with a patience of 5 epochs. Dropout (Srivastava et al., 2014) was applied in the hidden layers with rates of 0.5 and 0.25 for SHD and the other datasets, respectively. The available delay values are limited to remain within [0, 25] time steps for these datasets. The network is initialized without delays, i.e., all delays are equal to zero, and the input data were right padded accordingly for the network to allow delayed spikes. This exact setup was used for all experiments on all datasets presented in this study and implemented in PyTorch (Paszke et al., 2019). Our code is based on the SpArch implementation (Bittar and Garner, 2022). Table 1 shows an overview of the exact parametrization of the experiments, executed for this study. The setups for the SSC and GSC datasets were identical.

**Table 1:** Overview of the hyperparameters used in training for all datasets considered in this study.

**Dataset**	**Hidden layer size**	**Epochs**	**Batch size**	**Dropout rate**	**Lr weights**	**Lr delays**	**Initialization**	**Delay caps**
SHD	128	100	128	0.5	0.01	0.1	Xavier uniform	[0, 25]
SSC & GSC	512	100	32	0.25	0.001	0.01	Xavier uniform	[0, 25]

### 4.3 Analysis of the trainable adaptation parameters

In this section, we analyze the effects of the different parameter ranges for the main adaptation parameters *a* and *b* from the AdLIF neuron model, as presented in Equation (1). Our constrained Adaptive LIF (cAdLIF) model differs from the AdLIF model in two ways: we extended the available decay window for the membrane potential, the α parameter, for the model to be able to forget more quickly if needed. More importantly, we limited the available range of the *a* parameter, which allows the current membrane potential u[t] to influence the adaptation current w[t]. Similarly to adaptation by means of an adaptive threshold, this *a* parameter mainly limits spikes if the membrane potential was high in the past and thus provides a homeostatic mechanism, given that this *a* remains positive.

In Figure 3, we illustrate that for a possible negative *a*, the behavior of the adaptation current can lead to a non-controlled, chaotic spiking regime. In this figure, we count the number of spikes that are produced by a neuron, given a single fixed input spike train of 12 spikes, for different adaptation parametrizations of *a* and *b*. Whenever the produced number of spikes is higher than the number of input spikes, the model enters a chaotic regime in which a positive feedback loop could be activated and the neuron remains spiking, even without the presence of input spikes. Figure 3 shows that in the case for a negative *a*, where, apart from a region with a very high parameter *b*, the spike-triggered fraction of the adaptation current, the number of output spikes generated is very high. We therefore, unlike other works, chose to constrain in the top right quadrant with both positive *a* and *b* and hence the constrained AdLIF (cAdLIF) name for our neuron model. In this figure, the base LIF model can be found at the point, where *a* and *b* are equal to 0, and no adaptation current w[t] is produced.

### 4.4 Results

#### 4.4.1 Speech recognition datasets

In the first experiment, we investigated the effects of adding the trainable parameters to a basic LIF neuron model with similar initialization and the effects of updated parametrization boundaries for the adaptation parameters. Secondly, we added the trainable synaptic delays (models with d-) and evaluated the interplay of co-learning the adaptation and delays. The results in terms of classification accuracy, highlighting the individual contributions, are shown in Table 2, as tested on the three speech recognition datasets.

**Table 2:** Effect of the proposed enhancements in terms of accuracy, number of trainable parameters, and average spikes per neuron per sample, to a basic 2-hidden layer feedforward SNN model with a hidden layer size of 128.

		**LIF**	**AdLIF**	**cAdLIF**	**d-LIF**	**d-AdLIF**	**d-cAdLIF**	**fd-cAdLIF**
Accuracy (%)	SHD	84.49	91.67	94.19	92.57	93.40	94.85	93.40
	SSC	71.76	76.75	77.5	75.94	78.9	80.23	77.72
	GSC	86.21	93.97	94.67	89.81	95.3	95.69	95.05
# Parameters	SHD	37.9 k	38.7 k	38.7 k	74.8 k	75.8 k	75.8 k	38.7 k
	SSC	0.34 M	0.35 M	0.35 M	0.69 M	0.7 M	0.7 M	0.35 M
	GSC	0.30 M	0.30 M	0.30 M	0.60 M	0.61 M	0.61 M	0.30 M
#spikes/neuron	SHD	5.4	5.7	5.6	4.7	9,8	5.8	6.1
	SSC	6.6	14.6	3.9	7.7	9.2	5.2	6.2
	GSC	12.5	13.1	5.0	10.1	7.6	6.7	8.6

Given the limited size of the SHD validation set, the average accuracy over 10 runs is shown. The constrained AdLIF with trainable delays (d-cAdLIF) shows the best performance.

First, comparing the LIF model with the AdLIF and the cAdLIF neuron models on the SHD dataset, we observed that the average validation accuracy over 10 experiments is significantly increased by up to 7.2%. The cAdLIF model performs ~2.5% better than the AdLIF model, on average, which is in accordance with the empirical evaluation of adaptation parameters *a* and *b* in the analysis from Section 4.3. For the larger speech datasets, similar improvements are shown. Adding adaptation showed an increase of 5.0 and 8.1%, and the cAdLIF model further increased the accuracy with 0.75 and 0.7% for the SSC and GSC datasets, respectively. These results show the importance of trainable adaptation in SNN and adequate trainable neuron parameter boundaries. Similarly, the d-cAdLIF significantly outperforms the d-AdLIF and the d-LIF.

Secondly, the addition of trainable synaptic delays increases the average accuracy for the LIF, d-AdLIF, and cAdLIF neuron models by 8.1%, 1.7%, and 0.66% for the SHD dataset. The models with trainable synaptic delays are titled d-*SNN*. Comparably, enhanced results are shown for the SSC and GSC datasets. This shows that the extra provided capacity to utilize memory is beneficial for all speech recognition tasks and adds to the computational complexity already provided by the trained adaptation parameters. Additionally, co-learning both shows to further enhance our results. To validate the effectiveness of the proposed learning rule, we also trained an SNN with random heterogeneous, fixed (non-learnable) delays, called fd-cAdLIF. In this model, the delays were uniformly distributed within the pre-determined intervals ([0, 25]). The d-cAdLIF outperforms the fd-cAdLIF on all datasets.

Table 2 also shows the number of trainable parameters for all datasets and model configurations. The number of additional trainable parameters is limited when comparing the LIF with AdLIF and cAdLIF models as they only increase with the number of neurons in the SNN model not the synapses. Combined with the results in classification, these results clearly show the added value of trainable adaptation parameters for SNN models. Logically, the number of trainable parameters is doubled for the delay-enabled SNNs, as in that case, every synapse is characterized by both a delay and a weight value.

One of the properties that affects the efficiency of the SNN model, when deployed on neuromorphic hardware Yin et al. (2021), is the number of spikes that are required when processing a single sample in inference. We therefore analyze the number of spikes in the hidden layers to assess how the proposed improvements influence the total number of spikes in the SNN. To account for inter-experimental variance, we averaged the spike rates over 10 trials on the SHD dataset. The results are shown in Table 2.

We observed that the number of spikes is not significantly increased by the addition of the proposed adaptation method. For the SSC and GSC datasets, there is a decrease in the number of spikes, especially comparing the AdLIF and cAdLIF models. This shows that BPTT effectively ends up in regions where *a* or *b* < 0 and uncontrolled spiking behavior occur with the unconstrained AdLIF model. However, the synaptic delays, in general, result in an increase in the number of spikes in the SNN model.

#### 4.4.2 Experimental analysis on SHD

**Figure 4:**
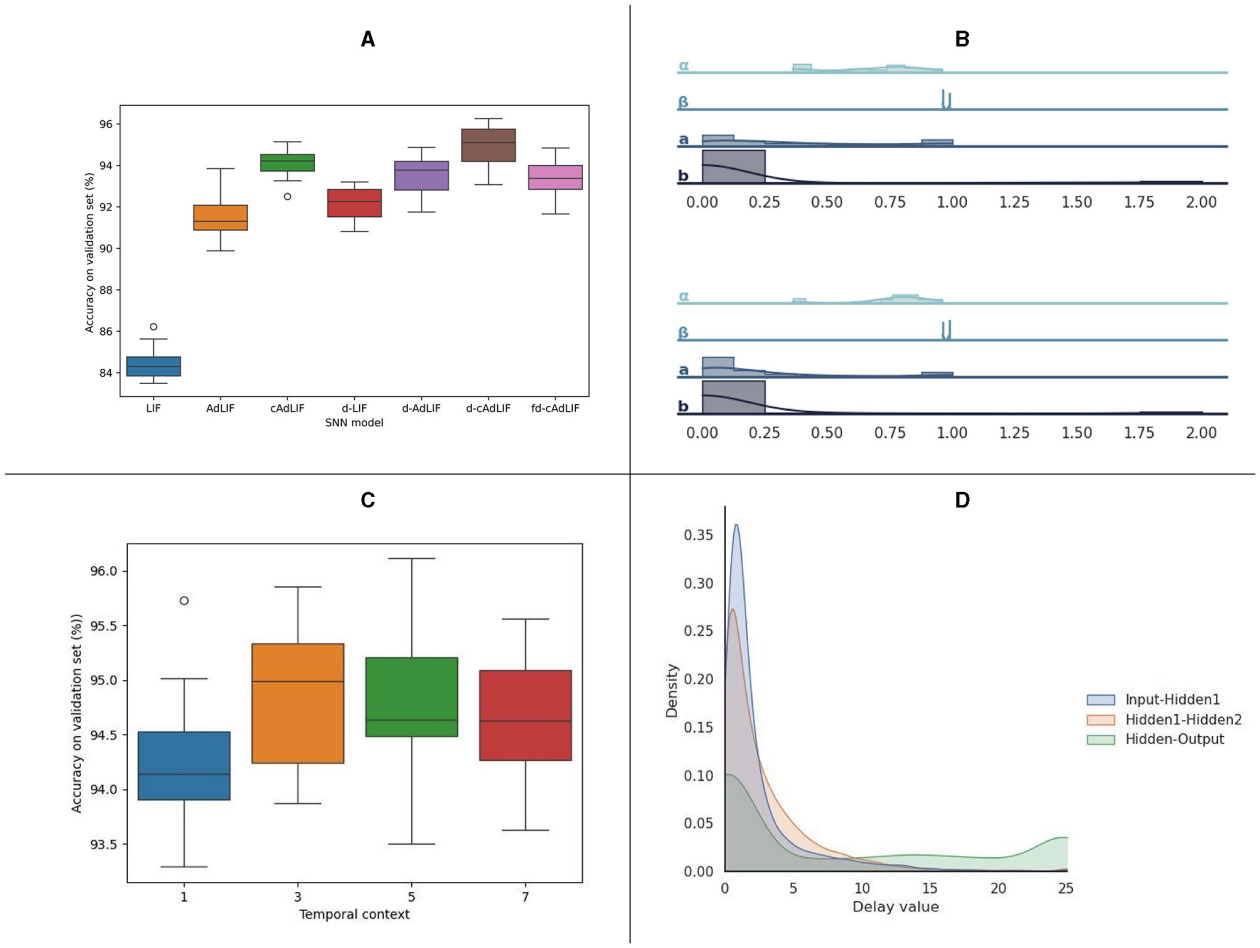
Analysis of the experiments on the SHD dataset. **(A)** Overview of the classification results for 10 independent trials for all evaluated neuron models. **(B)** Overview of the learned neuronal parameters in hidden layer 1 (top) and hidden layer 2 (bottom) for a d-cAdLIF model. **(C)** Experiments on the temporal delay parameter c, for 10 independent trials. **(D)** distribution of the learned synaptic delays per layer.

In Figure 4, we show a more detailed analysis of the SNN models which are trained on SHD. In Figure 4A, the distribution of the accuracy, as tested across 10 independent trials, is shown. The d-cAdLIF model clearly outperforms the cAdLIF model without synaptic delays and one with random fixed delays, showing the benefits of co-learning the adaptation and synaptic delays.

Figure 4B shows the distribution of the trained neuronal parameters. Interestingly, these are relatively similar for the two hidden layers. We note that specifically *a* and *b*, although uniformly initialized between their respective boundaries, show large proportion of near-zero elements.

In Figure 4C, we outlined the experiments on the temporal context *c*, which was used in training in the backward pass to determine the delay value updates. The results shown are for 10 independent d-cAdLIF neuron trials. We found that taking into account three timesteps in the past/future yielded the best average results. Finally, Figure 4D shows the distribution of the learned delay values for all three layers. We again note that many values are close to zero.

#### 4.4.3 Comparison to the state-of-the-art

An overview of the results of our experiments on the full cAdLIF model with synaptic delays across all speech recognition datasets is presented in Table 3. We compared our SNN model with state-of-the-art SNN solutions from the literature with a 2-hidden layer architecture and a corresponding state-of-the-art ANN model for each dataset, which is shown below the dotted line.

**Table 3:** Test accuracy on the SHD, SSC, and GSC datasets and comparison with state-of-the-art in SNN for 2 hidden layer feedforward models, ordered for the number of trainable parameters.

**Dataset**	**Model**	**Hidden size**	**# Parameters**	**Accuracy (%)**
SHD	Adaptive RSNN (Yin et al., 2021)	128	/	90.4
	**d-cAdLIF (ours)**	128	**0.076 M**	94.85 ± 0.64
	Axonal delays (Sun et al., 2022)	128	0.1 M	92.36
	Synaptic delays (Hammouamri et al., 2023)	256	0.2 M	95.07 ± 0.24
	DL256-SNN-DLoss (Sun et al., 2023a)	256	0.21 M	93.55
	RadLIF (Bittar and Garner, 2022)	1,024	3.9 M	94.62
	CNN (Cramer et al., 2020)	/	/	92.4
	Adaptive RSNN (Yin et al., 2021)	400	/	74.2
SSC	**d-cAdLIF (ours)**	512	**0.7 M**	**80.23** **±** **0.07**
	Synaptic delays (Hammouamri et al., 2023)	512	0.7 M	79.77 ± 0.09
	Synaptic delays^**^ (Hammouamri et al., 2023)	512	1.2 M	80.29 ± 0.06
	RadLIF (Bittar and Garner, 2022)	1,024	3.9 M	77.4
	GRU (Bittar and Garner, 2022)	512	/	79.05
	RSNN, LIF (Zenke and Vogels, 2021)	256	/	85.3
GSC	RSNN with SFA (Salaj et al., 2021)	2,048^*^	/	88.5
	**d-cAdLIF (ours)**	512	**0.61 M**	**95.69** **±** **0.03**
	Synaptic delays (Hammouamri et al., 2023)	512	0.7 M	94.91 ± 0.09
	RadLIF (Bittar and Garner, 2022)	512	0.83 M	94.51
	Synaptic delays** (Hammouamri et al., 2023)	512	1.2 M	95.29 ± 0.11
	GRU (Bittar and Garner, 2022)	512	/	94.32
	Transformer (Gong et al., 2021)	/	/	98.11

We report the average ± std for all results. Benchmark ANN results are shown below the dotted line. Our model is highlighted in bold.

^*^Recurrent SNN with a single hidden layer.

^**^Three hidden layers.

For the SHD dataset, the cAdLIF model with trained synaptic delays matches the state-of-the-art results of a recently proposed alternative method for training synaptic delays at just a fraction (less than half) of its number of trainable parameters and outperforms all other SNN and ANN methods on this dataset. Here, the cAdLIF model with trainable synaptic delays shows better performance than the current state-of-the-art models in SNN with a similar number of trainable parameters or less. Furthermore, one additional SNN model with three hidden layers was proposed by Hammouamri et al. (2023). This model achieves similar performance as ours on the SSC dataset 80.29 ± 0.06%, with an increase of ~45% additional trainable parameters (an additional hidden layer). This model only achieved 95.29 ± 0.11% on the GSC dataset, which is less than our SNN, which has just two hidden layers. Given the budget of trainable parameters, the proposed feed-forward SNN model even outperforms a non-SNN recurrent model (GRU) with the same preprocessing and moves closer to the performance of a large ANN model with transformer architecture.

## 5 Conclusion

In this study, we presented a novel SNN model, the cAdLIF with a novel temporal context-aware learning rule for synaptic delays. To the best of our knowledge, this is the first SNN model in which the synaptic delays are directly learned in coordination with the neuronal adaptation. Furthermore, we showed that (1) it is possible to co-learn synaptic weights, delays, and neuronal adaptation parameters at the same time and (2) co-learning these parameters proved to mutually benefit the optimization of all learned parameters as shown for three speech recognition datasets.

We highlighted that this co-optimization leads to state-of-the-art performance in SNN on all investigated datasets. The superior performance can be attributed to two additional features: (1) Training the synaptic delays enables a neuron in the SNN to explicitly correlate temporally distanced features and (2) The trained neuronal adaptation allows a greater variety in spike patterns, widening the feature space to be explored.

We showed that for a very simple architecture, a 2-hidden layer is fully connected to feedforward network; we are able to compete against and even outperform larger ANN models, with a limited number of trainable SNN parameters. The performance of the presented SNN model shows the promise of SNN research on tasks with rich temporal dynamics, and, in particular, research on biologically inspired extensions to existing SNN models.

When comparing with larger ANN models, the performance of the presented SNN model is lacking. A future step in our research is therefore to investigate how learning delays and adaptation parameters are influenced by the model architecture. More advanced architectures such as convolutional spiking neural networks or experimenting with the training recurrent synapses could further bridge the gap with ANNs. Another exciting avenue to be explored is the chosen neuron model. As the proposed cAdLIF model is a particular generalized integrate-and-fire model version (Gerstner and Kistler, 2002; Gerstner et al., 2014) and many more exist, there are various neuron model extensions available to be investigated. Future research will point out that to what extent, these will be useful for the application in SNN in the context of (1) their additional performance in terms of classification accuracy and (2) the additional complexity for their deployment on dedicated neuromorphic hardware.

Another point to explore is that our study includes a fixed maximal delay, which needs to be defined before training and requires fine-tuning. Including adjustable delay caps could benefit our approach. In future studies, we intend to investigate the effect of co-learning the synaptic weights and delays on more complex neuron models and model architectures and validate them on non-sound datasets.

## Data availability statement

The original contributions presented in the study are included in the article/supplementary material, further inquiries can be directed to the corresponding author.

## Author contributions

LD: Conceptualization, Data curation, Formal analysis, Funding acquisition, Investigation, Methodology, Project administration, Resources, Software, Supervision, Validation, Visualization, Writing – original draft, Writing – review & editing. LV: Conceptualization, Methodology, Writing – review & editing. WV: Project administration, Supervision, Writing – review & editing. IT: Conceptualization, Supervision, Validation, Writing – review & editing. SL: Funding acquisition, Project administration, Resources, Supervision, Writing – review & editing.
